# Safe ultrasound-guided percutaneous tracheostomy in eight steps and necessary precautions in COVID-19 patients

**DOI:** 10.1590/0100-6991e-20223202

**Published:** 2022-03-11

**Authors:** CARLOS AUGUSTO METIDIERI MENEGOZZO, CAROLINA CARVALHO JANSEN SORBELLO, JONES PESSOA SANTOS-JR, ROBERTO RASSLAN, SERGIO HENRIQUE BASTOS DAMOUS, EDIVALDO MASSAZO UTIYAMA

**Affiliations:** 1 - Hospital das Clínicas da Faculdade de Medicina da USP, Divisão de Clínica Cirúrgica III - Cirurgia Geral e Trauma - São Paulo - SP - Brasil

**Keywords:** Tracheostomy, Coronavirus Infections, Elective Surgical Procedures, Minimally Invasive Surgical Procedures, Traqueostomia, Infecções pelo Coronavírus, Procedimentos Cirúrgicos Eletivos, Procedimentos Cirúrgicos Minimamente Invasivos

## Abstract

Percutaneous tracheostomy has been considered the standard method today, the bronchoscopy-guided technique being the most frequently performed. A safe alternative is ultrasound-guided percutaneous tracheostomy, which can be carried out by the surgeon, avoiding the logistical difficulties of having a specialist in bronchoscopy. Studies prove that the efficacy and safety of the ultrasound-guided technique are similar when compared to the bronchoscopy-guided one. Thus, it is of paramount importance that surgeons have ultrasound-guided percutaneous tracheostomy as a viable and beneficial alternative to the open procedure. In this article, we describe eight main steps in performing ultrasound-guided percutaneous tracheostomy, highlighting essential technical points that can reduce the risk of complications from the procedure. Furthermore, we detail some precautions that one must observe to reduce the risk of aerosolization and contamination of the team when percutaneous tracheostomy is indicated in patients with COVID-19.

## RESUMO

A traqueostomia percutânea tem sido considerada o método padrão atualmente, sendo a técnica guiada por broncoscopia a mais realizada. Uma alternativa segura é a traqueostomia percutânea guiada por ultrassonografia, que pode ser feita pelo próprio cirurgião, evitando-se as dificuldades logísticas de disponibilidade de um especialista em broncoscopia. Estudos comprovam que a eficácia e a segurança da técnica guiada por ultrassonografia, comparada à guiada por broncoscopia, são semelhantes. Assim, é de suma importância que os cirurgiões tenham a traqueostomia percutânea guiada por ultrassonografia como alternativa viável e benéfica em relação ao procedimento aberto. Neste artigo, descrevemos oito passos principais da realização da traqueostomia percutânea ecoguiada, destacando pontos técnicos essenciais que podem reduzir o risco de complicações do procedimento. Ainda, detalhamos alguns cuidados que devem ser observados, com o intuito de reduzir o risco de aerolização e contaminação da equipe, quando a traqueostomia percutânea é indicada no paciente com COVID-19.

## INTRODUCTION

Tracheostomy is a procedure often indicated for patients admitted to intensive care units. Currently, the percutaneous route is considered the standard because it results in shorter procedure time and lower costs, in addition to a tendency towards a lower rate of complications such as surgical site infection and bleeding^1^ ^-^ ^3^.

The percutaneous procedure can be guided by anatomical palpation or by imaging tests such as bronchoscopy and ultrasonography^4^, which have shown superiority in relation to the first^5^ ^-^ ^8^. Bronchoscopy-guided percutaneous tracheostomy (BPT) is the most widespread method and has a safety profile like that of the ultrasound-guided technique (UPT)^5^ ^,^ ^9^.

In view of the COVID-19 pandemic and the recommendations of some societies against the use of bronchoscopy, UPT became more discussed and was incorporated in some services^10^ ^,^ ^11^. In services where tracheostomy is performed by thoracic surgeons trained in bronchoscopy, the performance of BPT can be facilitated. However, in hospitals where the bronchoscopy team works separately from the surgical team, there may be logistical obstacles to performing BPT. One of the advantages of UPT is that the surgeon who performs the tracheostomy, if properly trained, can manipulate the ultrasound device and perform the UPT, which can bring logistical and cost advantages, as it does not require an additional team member, the bronchoscopist. 

Thus, mastering the ultrasound-guided percutaneous technique involves mainly three important benefits: it offers patients the advantages of the minimally invasive percutaneous procedure, it guarantees a better safety profile than the technique based on anatomical palpation, and it eliminates the need for equipment and experience related to bronchoscopy.

Given the importance of this topic, this technical note aims to describe eight steps of the UPT, based on the literature and on the authors’ experience, from indication to postoperative evaluation, highlighting the main technical points that can reduce the risk of complications and increase the procedure’s efficiency. We also include the modifications that should be observed when performing UPT in patients with COVID-19, according to the protocol developed at our institution^11^, to increase the safety of the professionals involved, avoiding the use of bronchoscopy due to aerosolization risks.

### Step 1: Preoperative assessment of the cervical region

One of the main advantages of the ultrasound-guided procedure is the anatomical assessment of the cervical region. At this stage, the surgeon must identify the trachea, cricoid cartilage, tracheal rings, cervical vessels, and thyroid, preferably with a high-frequency linear transducer. The initial objective of this step is to exclude the presence of vessels located in the path of the tracheal puncture (midline), and to determine the location where the tracheal puncture can be safely and effectively performed, commonly between the second and third tracheal rings. It is noteworthy that some situations may be associated with difficulty in the procedure or even represent a contraindication to UPT, such as limitation of cervical hyperextention, cricofurcular distance smaller than 1.5-2cm, tumors and goiter, and the presence of larger vessels in the midline^4^ ^,^ ^12^.

We routinely measure the tracheal diameter (internal and external) and the distance between the skin and the anterior wall of the trachea in our procedures. The purpose of this step is to assist the physician in choosing the most suitable tracheostomy tube for the patient^13^ ^-^ ^16^. In general, we seek to use cannulas with an external diameter of at least 0.5-1cm smaller than the tracheal external diameter, to avoid cannulas of size disproportionate to the patient’s trachea, seeking to offer larger internal diameters (up to 9mm). It is not our routine to use cannulas with an external diameter greater than 13mm. Regarding the distance from the skin, we consider the use of adjustable tubes in patients with more than 2-2.5cm of distance between the skin and the anterior wall of the trachea if the dimensions of the conventional tube are disproportionate. It is noteworthy that the use of these ultrasonographic measures is largely due to our experience with the method, and to our knowledge there is no randomized study that routinely proves their benefit.

**Figure 1:**
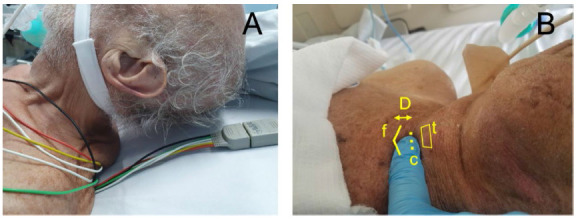
Some factors that may preclude ultrasound-guided percutaneous tracheostomy: Limited neck extension (A); and in B, and short distance (D) between the crichoid (c) and the sternal notch (f); Thyroid cartilage (t).

The distance between the cricoid cartilage and the sternal notch is an important preoperative factor and can be considered a relative contraindication when small, as it makes it difficult to position the transducer and to obtain a favorable ultrasound window for adequate tracheal puncture^12^ (Figure 1).

### Step 2: Positioning and sedation

**Figure 2:**
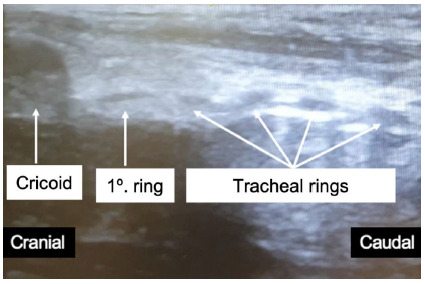
Sagital view of the trachea showing the crichoid cartilage and five tracheal rings.

The patient should be positioned similarly to a conventional tracheostomy. Our preference is for an interscapular pad and support for the occipital region, to increase the distance between the cricoid and the sternal notch and to superficialize and expose the trachea^12^ (Figure 2). The choice of sedatives is not within the scope of this technical note and must be individualized. An important detail is the use of neuromuscular blockers, which reduce the risk of accidental displacement of the orotracheal tube during traction and facilitate the introduction of the tracheostomy tube (step 6).

### Step 3: Choosing the puncture site

**Figure 3:**
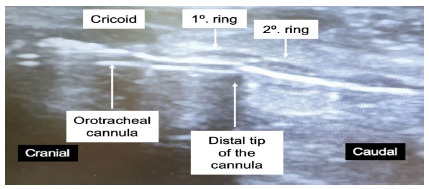
Sagital view of the trachea showing the orotracheal cannula represented by a double hyoerechoic line with acoustic shadowing. The tip of the cannula is located at the first tracheal ring.

All UPT steps are best performed using a high-frequency linear transducer. The tracheal tube should be inserted below the first tracheal ring to reduce the risk of tracheal stenosis. Most authors consider the position below the second tracheal ring to be ideal^4^ ^,^ ^12^ ^,^ ^17^ ^,^ ^18^. Ultrasonography allows the identification of tracheal structures and the planning of the puncture site with better accuracy than the technique based on anatomic palpation (APPT) (Figure 3)^7^.

### Step 4: Traction of the orotracheal tube

**Figure 4:**
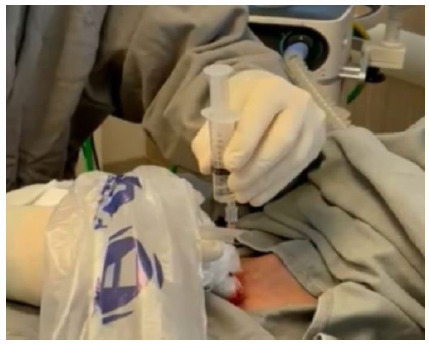
Centralized tracheal puncture with ultrasound guidance.

In UPT, traction of the orotracheal tube is performed under real-time sonographic visualization to position the tip at the height of the 1^st^ tracheal ring, reducing the risk of accidental extubation and, at the same time, preventing inadvertent perforation of the orotracheal tube or cuff during tracheal puncture. The orotracheal tube can be identified as a horizontal hyperechoic double line with posterior acoustic shadowing with the transducer in the longitudinal position (Figure 4).

In patients with COVID-19, tube traction should be performed after expiration and during an inspiratory pause to reduce the risk of aerosolization^19^, and ventilation should only be resumed after adequate cuff inflation^11^.

### Step 5: Puncture Centering

**Figure 5:**
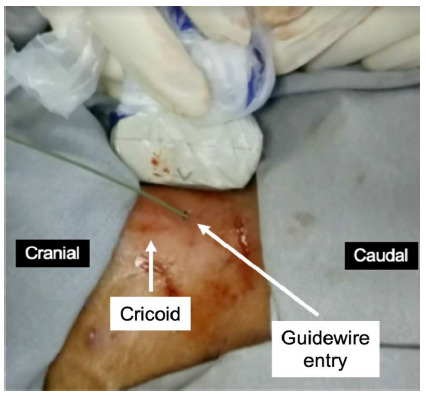
Sagital view of the trachea to identify the point where the guidewire enters the trachea.

The tracheal puncture must be ultrasound guided, ensuring that the needle is introduced in the midline, centered on the trachea^8^ (Figure 5). The use of ultrasonography results in more centralized punctures and a more successful first puncture^6^ and allows confirming the intratracheal positioning of the guidewire (Step 6). These are fundamental steps in the procedure as they can result in complications, such as injury to the trachea’s posterior wall and false path^18^ ^,^ ^20^.

### Step 6: Positioning of the guide wire

After puncture of the trachea, the guide wire is passed, whose position can be confirmed with ultrasonography. The surgeon must assess whether the guidewire entry is centered on the anterior wall of the trachea and confirm the height at which the wire entered the trachea. If the surgeon observes poor positioning of the guidewire, steps 5 and 6 must be repeated. After confirming the proper position of the guide wire, an incision is made in the skin, which can be longitudinal or transversal, and should have approximately 1.5cm.

In our institution, the first cases of UPT in COVID-19 patients involved stopping ventilation at the time of tracheal puncture^10^. After the first cases, our group changed the technique and started to stop ventilation after confirming the position of the guide wire, performed with occlusion of the puncture site, optimizing the apnea time.

In COVID-19 patients, we chose to perform a longitudinal incision before the orotracheal tube traction (step 4), to optimize the procedure time after tracheal puncture, at which point the risk of aerosolization increases. For the same reason, we opted for a slightly larger incision (1.5-2cm) to ensure that there is no difficulty in passing the tracheostomy cannula (step 8), at which time there is also a risk of aerosolization.

### Step 7: Tracheostoma dilation

Once the positioning of the guidewire has been confirmed, the progressive dilation of the trachea and the subcutaneous path must be continued. The most used dilation methods are probably the single conical dilator, derived from the technique described by Ciaglia^21^ (Figure 7), and Griggs^22^. Regardless of the kit, the principles of the procedure are the same. It is noteworthy that when identifying that the Griggs instrument has been inserted into the tracheal lumen, the instrument must be manipulated so that the tip is oriented towards the carina, taking care not to dilate the trachea with its tip oriented downward (posterior wall). This step should be performed with the patient in apnea in cases of COVID-19^10^ ^,^ ^11^.

**Figure 6:**
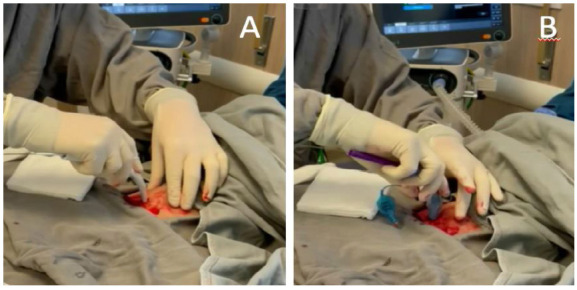
Tracheostoma dilation using the Ciaglia method (A). Tracheal cannula insertion through the dilated tract (B).

### Step 8: Passage of the cannula and assessment of placement and complications

Passing the tracheostomy tube using the guide wire contributes to reducing the risk of false paths and must follow the fundamental principles that are common to the conventional procedure (Figure 8). After positioning the cannula, confirmation that ventilation is adequate is preferably done with capnography. We routinely assess the presence of bilateral lung sliding with ultrasound after passage of the cannula, a useful method with high accuracy, especially in the absence of capnography. This method confirms the intratracheal positioning of the tube and excludes selectivity, a rare complication of tracheostomy, more accurately than auscultation^23^ ^,^ ^24^. We also use ultrasonography to assess at what height of the trachea the tube is positioned at the end of the procedure, confirming the proper positioning^25^.

We also routinely use ultrasound to exclude other complications, such as subcutaneous emphysema, hematomas, and pneumothorax. Despite the low incidence of these complications^24^ ^,^ ^26^, ultrasonography allows for early identification and implementation of therapeutic measures

## CONCLUSION

 UPT is a well-established technique with results similar to BPT. In the context of the COVID-19 pandemic, the risks associated with bronchoscopy have resulted in a reduction in the performance of BPT, even where tracheostomies are performed by thoracic surgeons trained in bronchoscopy. The incorporation of ultrasound in percutaneous tracheostomy by the surgeon is feasible, offering patients the benefits of the percutaneous technique without the possible logistical difficulties and costs associated with bronchoscopy, especially in services without thoracic surgeons qualified for this procedure. Furthermore, it is a feasible technique in cases of COVID-19 if the essential surgical steps are properly performed when there is higher risk of aerosolization. UPT is, therefore, a technique that should be incorporated into the therapeutic arsenal of surgeons who perform tracheostomies.
